# Responding to sustained poor outcomes in the management of non-communicable diseases (NCDs): an “incident control” approach is needed to improve and protect population health

**DOI:** 10.1186/s12889-019-6881-3

**Published:** 2019-05-16

**Authors:** Julia Knight, Matthew Day, John Mair-Jenkins, Chris Bentley, Ben Anderson, Fu-Meng Khaw

**Affiliations:** 1 Public Health England East Midlands Centre, Seaton House, City Link, Nottingham, NG2 4LA UK; 2 School of Health and Related Research, The University of Sheffield, 30 Regent St, Sheffield, S1 4DA UK; 3 University of Leicester Medical School, University of Leicester, Centre for Medicine, Lancaster Rd, Leicester, LE1 7HA UK

**Keywords:** Non-communicable disease, Response, Poor outcomes, Parity, Incident control approach

## Abstract

In 2017 Public Health England were asked to assist with investigating why 1-year cancer survival rates appeared lower than expected in a local area. We identified 50 premature deaths that surveillance data suggested we would not expect. These deaths highlighted a gap in recognising and responding to this kind of systematic non communicable disease (NCD) outcome variation. We hypothesise that the lack of a universally agreed systematic response to variations is not only counter-intuitive, but wholly unacceptable where non-communicable diseases (NCDs) rather than infectious diseases have become the leading causes of illness and death worldwide. In the United Kingdom (UK) alone over 89% of mortality in 2014 was attributable to NCDs. We argue that a new approach is urgently needed to turn the curve on NCD outcome variation to protect and improve the public’s health. We set out a definition of an NCD “incident” and propose a phased approach that could be used to respond to local variation in NCD outcomes.

Establishing parity of response for local variations in NCD outcomes and CD control is critically important. Although evidence shows that prevention and early intervention will make the biggest difference to NCD incidence, collective local whole health economy response, exploiting the wealth of surveillance data in real time, needs to be at the heart of responding to variations in NCD outcomes at a population level. We argue that local and national public health agencies should mandate a standardised ‘incident’ response to significant changes in outcomes from NCD to mitigate and reduce the loss of quality life.

## Key messages

3 things we want to happen:

- Agencies to have parity of response for local variations in NCD outcomes and CD control.
- To build body of evidence about how proposed approach works across investigations into different NCD responses.
- Make legislative changes to PH act to reflect the growing need to better protect the public from variations in NCD outcomes.

## Background

What happens if there are 50 unexpected deaths in a 5 year period attributable to a communicable disease (CD)? In a country with a developed public health system this scenario is almost inconceivable as actions would have been taken to prevent these deaths soon after the incident was recognised. Resources are quickly mobilised; supported by systematic surveillance, well-practiced incident response systems and consensus of what constitutes an incident/ outbreak; all of which is mandated in national [1, 2] and international legislation [3].

**Fig. 1:**
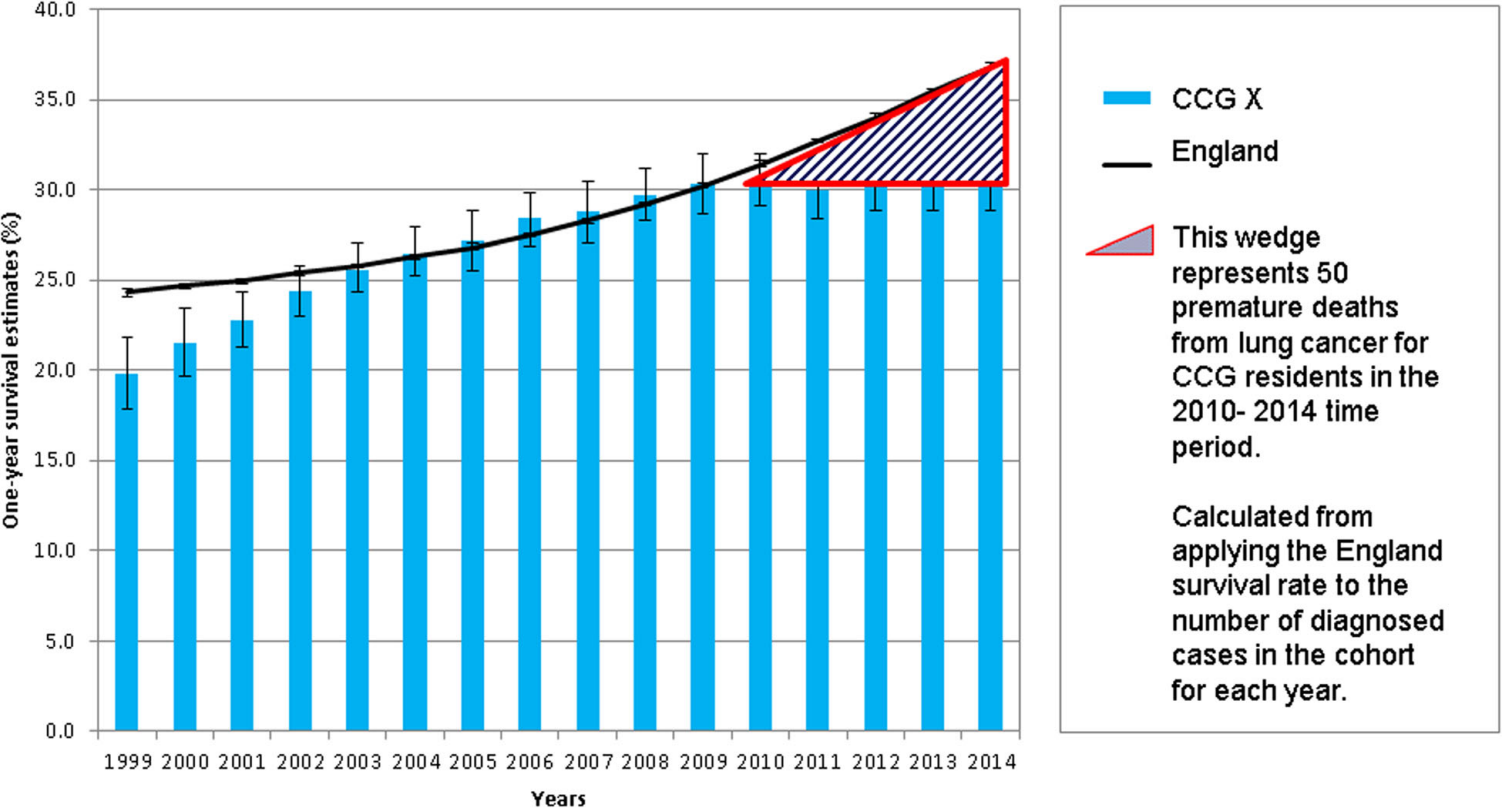
Variation in 1-year lung cancer survival rates between a CCG and the England average [24]

Now consider the same scenario of 50 premature deaths attributable to a non-communicable disease (NCD), where surveillance data shows we would not otherwise expect this (Fig. 1). There is currently no accepted standard response to such an incident.

There are two inherent prejudices between NCD and CD control that may have limited previous thinking on the parity of approach:

1. It can be argued that NCD outcome variation can only harm relatively small numbers of people affected at local level over short periods of time whereas localised CD outbreaks can escalate exponentially, and in extreme cases turn into national and international outbreaks harming thousands of people, trade [3] and generating high profile media coverage and, public concern if not rapidly controlled.
2. The aetiologies of NCDs are wide ranging, complex, and, lead time from exposure to diagnosis can be decades [4–6]. These are often chronic health issues for individuals as opposed to acute CDs. This may mask that variation in outcomes for NCDs may be an acute issue for a local health system.

The lack of a systematic response to NCDs may have increased variation in outcomes that is readily observed within populations [7–9]. The failing of health and public health systems to effectively and efficiently tackle variation in outcomes [6] is at odds with the significant achievements public health agencies have demonstrated in reducing the burden of CDs [10].

## Main text

We propose six changes, necessary to remove the inherent prejudices and test our proposals to achieve parity of response with the management of outbreaks of CDs.

### CHANGE 1: Remove the ‘strategy paradox’ for NCDs

Long term NCD strategies for international or national geographies guide the implementation of approaches to improve NCD outcomes. In England successive plans have been developed to tackle the major NCDs. Paradoxically, despite their aims, these strategies fail to address or respond to locally changing patterns of disease. Referring back to our opening scenario, and Fig. 1, one of the most recent national plans is the Cancer Strategy in England. This has the aim of “improving survival rates and saving thousands of lives” [11] and includes 96 recommendations and the formation of a multitude of working groups. However there is no recommendation for systematic surveillance, control systems, and, response to reduce adverse changes in local cancer outcomes. Unlike CD guidelines [12], NCD strategies are not prescriptive or do not mention the steps local areas should take to respond to variation in outcomes. We argue that these are fundamental to achieve parity with CD control.

### CHANGE 2: Translate surveillance data on NCDs into meaningful local action

In CD management, streamlined passive surveillance by diagnostic and public health laboratories, under the leadership of a national public health agency, has been the hallmark for improving responses [1, 2] and reducing burden. This system, coupled with local intelligence, often leads into the initiation of further investigations e.g. active and enhanced surveillance along with incident/outbreak identification and response. It is an approach that is supported by the standards set out in the International Health Regulations (IHR) 2005 [3].

In the United Kingdom (UK), variations in NCD outcomes are observed in a number of Government [13], National Health Service (NHS) [14] and, public health [15] sources. To combine and translate NCD surveillance data into meaningful local action, the information needs to be understood from a whole local health economy perspective so that assumptions can be challenged, root causes understood, actions allocated and, ultimately step changes in improvements made. It is unsatisfactory to assume that population demography is the only cause of variation without fully testing this hypothesis but at present, no centrally co-ordinated effort has been made to standardise an approach. In our lung cancer investigation alone, we examined six datasets published by four separate agencies. We recognise that additional datasets would be useful but are neither publicly available nor standardised.

We acknowledge that globally, surveillance systems in some low and middle income countries are less well established. Whilst this will hinder progress on this step change in the short term, the call for parity of approach for NCD incidents (in comparison to CD incidents) is applicable as health system architects consider how to design and embed surveillance systems to understand and improve public health in the future.

### CHANGE 3: Develop accountability and ownership of local NCD responses

The ‘who is responsible for action’ related to improving NCD outcome and reducing variation can also result in complexity. For example, as an output of the Cancer Strategy in England [12], several recommendations focussed on increasing data availability, ensuring audit of deaths related to treatment and, holding local health bodies in England to account for 1-year survival outcomes. At the time this was heralded as a “transformational” change for cancer services. It was regarded as a driver “for Clinical Commissioning Groups (CCGs) to work across all relevant organisations … to improve survival rates” [16]. However, this performance management approach, in which there is the potential for a culture of either commissioner or provider blame [17, 18], is at odds with the culture of a CD incident response [12]. In the latter, the accountability for patient outcomes (outside of a specific clinical setting) are not inherently owned by one organisation of the NHS, and the system response is collective, led by public health specialists and is focused on improving outcomes and quality of care [1, 12]. We believe that local accountability and ownership of NCD responses should be embedded into developing place based health systems and recognise the opportunity that the ongoing emergence of new care models provides to achieve this.

### CHANGE 4: Agree a common definition of a NCD ‘incident’

Unlike with CD [1, 2] there is lack of clarity in both national strategies and the literature about what might constitute an ‘incident’ relating to variations in NCD outcomes. A working definition would help address the segregation and silo working [17, 18] currently inherent in NCD response and provide a common language to facilitate collaborative action (Table 1).

**Table 1:** Defining a health protection and a NCD “incident”

In the UK, a health protection incident may be defined as [12]: - “an incident in which two or more people experiencing a similar illness are linked in time or place” - “a greater than expected rate of infection compared with the usual background rate for the place and time where the incident has occurred” - “a single case for certain rare diseases”	A NCD “incident” definition proposed by the authors as comprising: - a significant and sustained step changed deterioration in population outcomes in comparison to the baseline trend or comparator And - the individuals or populations have similar conditions or have accessed the same health system and are linked in time or place And there is - potential for single cause or focus of variation

In defining a NCD “incident” the focus is on understanding changes in outcomes in a local area over time. Solely having high rates of mortality from lung cancer in comparison to an average would not qualify, nor would having a historically higher than expected rate of lung cancer in the local area. Instead, there would need to be a significant and sustained change in (e.g. lung cancer) outcomes in a defined area over a successive time period.

Additionally, as stated in Table 1 with the phrase “potential for single cause or focus of variation”, there would need to the possibility of a common origin of variation for changes in NCD outcomes to be recognised as an “incident”. Potential foci could be common demographics of patients affected, a common healthcare organisation or service, a specific pathway of care or even potentially the same healthcare professionals involved in delivering care. This would be necessary to warrant public health investigation given the opportunity cost and potential unintended consequences of investigating poorly defined NCD incidents.

### CHANGE 5: Implement a standardised incident control response to investigate NCD outcome variation

Once we have defined an NCD incident, how do we respond rapidly, locally? We argue that the response could take the same phased approach as a CD response (Table 2) and that lessons can be adopted from the standard guidelines used by health protection teams across the UK [12]. It is suggested that the leadership and oversight of this should be owned by a statutory body.

**Table 2:** Phased approach to respond to local variation in CD vs. NCD outcome variations

Health Protection guidelines [12]	Area of response	*Proposed* Noncommunicable Disease guidelines
“Initial investigation to clarify the nature of the outbreak [refer to Table 1] begun within 24 h Immediate risk assessment undertaken and recorded following receipt of initial information”	Incident recognition	*Use routinely collected data for surveillance and early recognition of change in outcomes. Investigate potential incident of sustained or step change with possible single cause or focus of variation*
“Decision made and recorded at the end of the initial investigation regarding outbreak declaration and convening of Outbreak Control Team (OCT)”	Incident declaration	*Decision made and recorded at the end of the initial investigation regarding incident declaration and convening of Incident Control Team (ICT) from appropriate partner organisations*
“Outbreak Control Team held as soon as possible and within three working days of decision to convene all agencies/disciplines involved in investigation and control represented at OCT meeting” “Roles and responsibilities of OCT members agreed and recorded” “Lead organisation with accountability for outbreak management agreed and recorded”	Incident Control Team	*ICT held as soon as possible and within ten working days of decision to convene all agencies/disciplines involved in investigation and control represented at ICT meeting* *Roles and responsibilities of ICT members agreed and recorded* *Lead organisation with accountability for incident management agreed and recorded*
“Control measures documented with clear timescales for implementation and responsibility” “Case definition agreed and recorded” “Descriptive epidemiology undertaken and reviewed at OCT. To include: number of cases in line with case definition; epidemic curve; description of key characteristics including gender, geographic spread, pertinent risk factors; severity; hypothesis generated” “Review risk assessment in light of evidence gathered” “Analytical study considered and rationale for decision recorded” “Investigation protocol prepared if an analytical study is undertaken”	Incident investigation and control	*Urgent control measures indicated from initial investigation agreed and implemented* *Outcome deterioration definition agreed and additional data to support investigation sourced from ICT members/partner organisations.* *Descriptive epidemiology of routine and additional data undertaken and reviewed at ICT to aid hypothesis generation. To include: outcome trend over time; description of key characteristics of cases including age, sex, access to health care, pertinent risk factors (*e.g. *late stage cancer diagnosis);severity (*e.g. *estimated impact on mortality);* *Review/implement control measures and public health interventions in light of evidence gathered* *Analytical study considered and investigation protocol prepared if an analytical study is undertaken* *Agree defined markers of point where control has been re-established*
“Communications strategy agreed at first OCT meeting and reviewed throughout the investigation” “Absolute clarity about the outbreak lead at all times with appropriate handover consistent with handover standards”	Communications	*Communications strategy agreed at first ICT meeting and reviewed throughout the investigation.* *Absolute clarity about the lead organisation at all times and point of closure of incident*
“Final outbreak report completed within 12 weeks of the formal closure of the outbreak” “Report recommendations and lessons learnt reviewed within 12 months after formal closure of the outbreak”	End of Incident	*Final outbreak report completed within 12 weeks of the formal closure of the incident* *Report recommendations and lessons learnt reviewed within 12 months after formal closure of the incident*

Defining standard, evidence informed tools that can be used by incident control leads and stakeholder partners to ensure control methods are employed in a timely fashion to minimise the impact of variation in NCD outcomes should be a priority. The authors recognise that the decision to formally end an NCD incident response is not yet defined; further work is needed to understand the necessary pre-requisites.

### CHANGE 6: Consider legislative response to ensure parity for NCDs

In the UK the Public Health (Control of Disease) Act 1984 [1] enshrined in law the requirement for a response to outbreaks of infections. This resulted in successive guidance on standards, shaped by historical failures, for the recognition, declaration, team response, investigation, control, communication and, end of incident [12].

The call for an incident control team meeting, or its equivalent, is not routine practice or a statutory responsibility in managing variation in NCD outcomes. This can result in de-prioritisation of the issues amongst stakeholders across the health sectors, which further exacerbates the population health risk. But, merely having a statutory duty may not be enough. The NHS has a statutory “duty” to “consider” reducing health inequality [19] but so far this has not, as yet, resulted in an entirely equitable system [20]. Considering need for legislative parity to mandate robust surveillance and mechanisms for response to variation in NCD outcomes is essential if society is to get serious about responding to incidents of NCD.

## Discussion

In 2013, the Department of Health (as it was at that time) published the “Living Well for Longer…” policy paper. Within this, Jeremy Hunt, (Secretary of State for Health at the time) called on “all those involved across the health and care system and beyond” to “play their part and work together” … “to determine what they should be doing to support their local communities to live longer, healthier lives” [21]. Jeremy Hunt stated it was “time to be bold and ambitious for health”. Despite efforts over the last 6 years to improve the transparency of data [22] and develop new models of health and care delivery [23], we argue that without concentrated effort and resource to define an NCD incident and standardise a response using evidence informed tools we will not be able to achieve the great strides in health outcome improvements for NCD that have been accomplished by our counterparts working in CD control.

## Conclusion

The morbidity, mortality and health inequalities related to NCD are increasing both domestically and globally. Whilst evidence shows that prevention and early intervention will make the biggest difference to NCD incidence, we believe that collective local whole health economy response, exploiting the wealth of surveillance data in real time, needs to be at the heart of responding to variations in NCD outcomes. This requires a cultural shift in historical approaches. We recognise that the time frame for defining and hence responding to an NCD incident is ambiguous at present. However, as highlighted in our recent investigation of variation in 1 year survival post cancer diagnoses, each year of delayed or ‘slow burn’ investigation will affect the survival and outcomes of a current cohort in the short and longer term. We argue that the ambition should therefore be for local and national public health agencies to determine their interpretation of ‘significant and sustained’ changes in population outcomes from NCD and mandate a standardised ‘incident’ response to mitigate and reduce the loss of quality life. The development of evidence-informed and pragmatic guidelines are required to standardise this approach and test its merit. Additionally, the specialist workforce required to make this step change must be considered. It may be sensible to initially focus on how definitions and guidance could look for such ‘incidents’ related to the main causes of premature death, perhaps CVD and cancer. We welcome suggestions to develop the definition for “NCD incident and response” to ensure that we can collectively rise to the challenge of protecting our patients, and populations from variations in outcomes from NCD.

## Abbreviations

CCG: Clinical Commissioning Groups
CD: Communicable disease
ICT: Incident Control Team
NCD: Non-communicable disease
NHS: National Health Service
OCT: Outbreak Control Team
UK: United Kingdom
